# Suprachiasmatic nucleus functional connectivity related to insomnia symptoms in adolescents with major depressive disorder

**DOI:** 10.3389/fpsyt.2023.1154095

**Published:** 2023-05-16

**Authors:** Lingling Cao, Ruohan Feng, Yingxue Gao, Weijie Bao, Zilin Zhou, Kaili Liang, Xinyue Hu, Hailong Li, Lianqing Zhang, Yang Li, Lihua Zhuo, Guoping Huang, Xiaoqi Huang

**Affiliations:** ^1^Department of Radiology and Huaxi MR Research Center (HMRRC), Functional and Molecular Imaging Key Laboratory of Sichuan Province, West China Hospital, Sichuan University, Chengdu, China; ^2^Department of Radiology, Sichuan Mianyang 404 Hospital, Mianyang, China; ^3^Department of Radiology, Sichuan Mental Health Center, The Third Hospital of Mianyang, Mianyang, China; ^4^Research Unit of Psychoradiology, Chinese Academy of Medical Sciences, Chengdu, China; ^5^Department of Psychiatry, Sichuan Mental Health Center, The Third Hospital of Mianyang, Mianyang, China

**Keywords:** major depressive disorder, suprachiasmatic nucleus, functional connectivity, adolescent, insomnia

## Abstract

**Background:**

Insomnia is a commonly seen symptom in adolescents with major depressive disorder (MDD). The suprachiasmatic nucleus (SCN), which is the circadian rhythm regulation center, plays a crucial role in the regulation of sleep-wake circulation. Nevertheless, how SCN function contributes to the exact neural mechanisms underlying the associations between insomnia and depressive symptoms has not been explored in adolescents. In the current study, we aimed to explore the relationship between SCN functional connectivity (FC) and insomnia symptoms in adolescents with MDD using a seed-based FC method.

**Methods:**

In the current study, we recruited sixty-eight first-episode drug-naïve adolescents with MDD and classified them into high insomnia (MDD-HI) and low insomnia (MDD-LI) groups according to the sleep disturbance subscale of the Hamilton Depression Rating Scale (HAMD-S). Forty-three age/gender-matched healthy controls (HCs) were also recruited. SCN FC maps were generally for all subjects and compared among three groups using one-way ANOVA with age, gender and adjusted HAMD score as covariates. We used partial correlations to explore associations between altered FC and clinical symptoms, including sleep quality scores.

**Results:**

Adolescents with MDD showed worse sleep quality, which positively correlated with the severity of depression. Compared to MDD-LI and HCs, MDD-HI adolescents demonstrated significantly decreased FC between the right SCN and bilateral precuneus, and there was no significant difference between the MDD-LI and HC groups. The HAMD-S scores were negatively correlated with bilateral SCN-precuneus connectivity, and the retardation factor score of HAMD was negatively correlated with right SCN-precuneus connectivity.

**Conclusion:**

The altered FC between the SCN and precuneus may underline the neural mechanism of sleep-related symptoms in depressive adolescents and provide potential targets for personalized treatment strategies.

## 1. Introduction

Major depressive disorder (MDD) which is characterized by sadness, loss of interest, disturbed sleep, or loss of appetite (1) affects approximately 322 million people worldwide and is the single largest contributor to years lived with disability (2). In China, one recent study reported that a significant increase in the prevalence of mental disorders in children and adolescents (3), with a prevalence of depression of approximately 3% among 73,992 children and adolescents aged 6–16 years, and the prevalence increased with age (4). Adolescence is a critical period for brain structural changes and psychological development. Adverse outcomes associated with depression in adolescents include recurrence of depression, comorbidity with other psychiatric disorders, and lasting impairment in sociability and learning abilities (5). Therefore, the identification and early intervention of depression in adolescence is particularly important.

Insomnia is one of the diagnostic criteria for MDD according to the DSM-5 (6), and previous studies have shown that adolescents with depression are more likely to have sleep disturbances than adults (7). It had been suggested that adolescent depression is marked by an increased incidence of insomnia and dramatic changes in sleep patterns (8, 9). Meanwhile, sleep duration of less than 6 h per night and high academic pressure were both associated with an increased risk of depressive symptoms in adolescents (10). Adolescents with comorbid mood symptoms and sleep problems tend to have more severe depressive symptoms and higher rates of suicides and self-harm, they tend to respond less well to antidepressant treatment, and sleep disturbances often lingering for longer periods even when symptoms of depression resolve (8, 11, 12). Recognizing the heterogeneity of depression and analyzing specific symptoms and their causal relationships is the first step toward personalized treatment of depression (13, 14). Therefore, it is worthwhile to explore the neural mechanisms of depression-related sleep disorders.

The suprachiasmatic nucleus (SCN), as the circadian rhythm regulating center of mammals (15, 16), plays an important role in regulating sleep-wake (15). The human SCN is a cluster of approximately 10,000 tightly interconnected neurons located above the bilateral optic chiasm along the midline of the brain near the ependymal lining of the third ventricle (17). It receives information about the ambient light levels via intrinsically photosensitive retinal ganglion cells (ipRGCs) and relays circadian information to the rest of the brain through synaptic and hormonal mechanisms; in this way, they coordinate the daily cycles of physiology and behavior (18–20). Disruptions of the SCN cause animals to lose the circadian rhythm; however, circadian rhythm can be restored by grafts of neonatal SCN tissue into SCN-ablated rodents (21). In addition, circadian alterations (e.g., light pollution, shift work, seasonal changes, jet lag or social jet lag) have been associated with depression or depressive-like behavior in humans (18). Abnormalities in the sleep-wake cycle, physiological rhythms such as hormones, and body temperature are often observed in patients with depression (22). Moreover, depressive symptoms varied over a 24 h cycle (23). Thus, dysregulation of internal SCN mechanisms may be the main neural mechanism of insomnia in depressed patients.

More recently, psychoradiology has emerged as an effective method for exploring the relationship between brain structure or function and behavior (24), and several lines of evidence have suggested that functional abnormalities in the brain SCN may play an important role in the relationship between depression and insomnia symptoms. One study reported that decreased functional connectivity (FC) between the right SCN and the superior temporal gyrus is correlated with diurnal mood variation (DMV) symptoms in patients with depression (25). Most MDD patients with DMV tend to have early sleep disorders, which manifest as abnormal amplitude of low-frequency fluctuation (ALFF) of the orbital superior frontal gyrus and increased FC between the left SCN and the superior limbic gyrus (23). Abnormal FC between the SCN and the right middle frontal gyrus, right fusiform gyrus, right lingual gyrus and left calcarine sulcus was observed in MDD patients with early wakening (26). Nevertheless, the MDD patients included in the studies mentioned above were all medicated, so the results could be influenced by the effect of medication. More importantly, previous studies all focused on adult sample, and no study has investigated the relationship between SCN FC and sleep quality in adolescents with MDD.

Therefore, this study recruited a relatively large and homogeneous sample of first-episode drug-naïve patients without other comorbidities to rule out the confounding effect of drugs and comorbidities. The aim of the current study was to investigate SCN FC alterations in the whole brain of adolescent patients with depression and their relationship with sleep quality. For this purpose, we hypothesized that there are abnormalities in the intrinsic function of the SCN in adolescents with depression and that these abnormalities would be associated with the sleep symptom severity of adolescent MDD.

## 2. Methods

### 2.1. Participants

A total of 68 drug-naïve adolescent patients with first-episode depression and 44 age-, sex-, and education-matched healthy controls (HCs) were recruited from the Third People’s Hospital of Mianyang, Sichuan, China. All patients were diagnosed with depression by two experienced professional psychiatrists using the Structured Clinical Interview (SCID) based on the Diagnostic and Statistical Manual for Mental Disorders, Five Edition (DSM-5). The inclusion criteria were as follows: (1) meeting the DSM-5 diagnostic criteria for MDD; (2) 12–18 years old; (3) total score of Hamilton Depression Scale (HAMD) more than 8; (4) right handedness; (5) no history of medication or other antidepressant treatment; and (6) no comorbidity with other mental disorders (e.g., bipolar disorder, attention deficit/hyperactivity disorder).

The HCs were recruited by poster advertisements, and the HCs were interviewed using the non-patient edition of the SCID. Common exclusion criteria for all subjects were family history of neurological and psychiatric disease, presence of substance abuse or dependence, and any contraindications to MRI scanning. One HC subject was excluded due to head movement correction and quality control. Finally, 68 MDD patients and 43 HC individuals were included for further statistical analysis.

The Ethics Committee of the Third People’s Hospital of Mianyang approved the study. Written informed consent was obtained from all participants and their legal guardians before participation in the trial. The subsets of data used in this paper have been used in previous studies (27, 28).

### 2.2. Clinical measures and subgroup division

The depressive symptoms of patients were evaluated based on the 24-item Hamilton Depression scale (HAMD-24) (29), with higher total score indicating more severe symptoms. The HAMD sleep disorder subscale (HAMD-S) was used to evaluate the patients’ insomnia symptoms according to the score of items 4 (inability to fall asleep), 5 (night waking), and 6 (waking too early) of the HAMD-24. The critical value of the severity of insomnia symptoms in depression as a HAMD-S score of 3, so that patients were divided into MDD with higher insomnia symptoms (MDD-HI, HAMD-S score > 3) and MDD with lower insomnia symptoms (MDD-LI, HAMD-S score ≤ 3) (30). Finally, to minimize the effect of sleep disturbance on depressive symptom scores, we calculated the adjusted HAMD scores by removing the sleep subscale. HAMD-s score were less than 3 score in all HCs.

In addition, for all subjects, we used the 14-item Hamilton Anxiety Scale (HAMA-14) (31) to assess anxiety symptoms and the Pittsburgh Sleep Quality Index (PSQI) (32) scale to assess the seep quality in the past month.

### 2.3. MRI data acquisition

A 3.0-T Siemens MR system (SKYRA) equipped with a 20-channel phased-array head coil was used for the MRI scan. The resting-state functional magnetic resonance imaging(rs-fMRI) data of the whole brain were acquired by a gradient echo-planar imaging sequence. The parameters included: repetition time (TR) = 2,000 ms, echo time (TE) = 30 ms, number of axial slices = 35, slice thickness = 4 mm, slice gap = 0.2 mm, flip angle = 90^°^, image matrix size = 64 × 64, voxel size = 3.75 × 3.75 × 4 mm^3^, and field of view (FOV) = 240 × 240 mm^2^. The rs-fMRI lasted 8 min in total, and 255 volumes were acquired for each participant. High-resolution T1-weighted anatomical images were acquired using a magnetization prepared rapid gradient echo sequence (TR = 1,900 ms, TE = 2.25 ms, 176 slices, slice thickness = 1 mm, flip angle = 9^°^, matrix size = 256 × 256, voxel size = 1 × 1 × 1 mm^3^). All participants were instructed to remain relaxed and awake with their eyes closed and without directed thoughts during scanning.

### 2.4. Data preprocessing

The preprocessing of functional images was conducted using the Data Processing and Analysis for Brain Imaging toolkit (DPABI^1^) (33) based on MATLAB R2018b. Specifically, the first 10 volumes of scanned data for each participant were discarded to ensure signal stabilization. The remaining images were corrected for acquisition time intervals between slices and then rearranged to the first volume for head motion correction. To reduce the influence of head motion, we regressed out six head motion parameters, six head motion parameters one time point before, and the corresponding 12 squared items (based on the Friston 24-parameter model). Nuisance signals from the white matter and cerebrospinal fluid (CSF) were also regressed to reduce the effects of physiological artifacts. The effects of linear trends were also removed. After all these corrections, the images were spatially normalized into the standard Montreal Neurological Institute (MNI) space and resampled to 3 × 3 × 3 mm^3^. We smoothed the data using an isotropic Gaussian kernel with an 8-mm full width at half maximum. Finally, bandpass filtering (0.01–0.08 Hz) was performed.

The mean framewise displacement (FD) was calculated as a measure of the microscale head motion for each participant. Participants with a mean FD < 0.2 mm were included.

### 2.5. Seed-based FC analysis

The bilateral SCN seed regions were defined as spheres with a 2-mm radius and centered at Talairach coordinates: (*x* = 3, *y* = 5, *z* = −8) for the right SCN and (*x* = −2, *y* = 5, *z* = −8) for the left SCN (17). Seed-based rs-FC analysis was performed using the RESTPlus software.^2^ The time series were first extracted for each seed region. We calculated the Pearson correlation between each seed and the remaining voxels of the brain to acquire whole-brain FC maps. The correlation coefficients were then converted to z values using the Fisher r-to-z transformation to improve normality.

### 2.6. Statistical analysis

#### 2.6.1. Comparison of demographic and behavioral data

Statistical analysis was performed using the Statistical Package for the Social Sciences (SPSS, version 19.0, Inc., Chicago, IL, United States). One-way analysis of variance (ANOVA) and the chi-square test were used to compare the demographic and clinical data among the three groups. Specifically, one-way ANOVA was used to compare age, education and HAMD scales, HAMA scales, and PSQI scales, while chi-square tests were used for sex. The threshold of statistical significance was set at *p* < 0.05 (two-tailed).

Pearson correlation was used to analyze the associations between HAMD, HAMA, and PSQI scores.

#### 2.6.2. Comparison of SCN FC maps

The SCN FC maps of the MDD-HI, MDD-LI and HC groups were compared using one-way ANOVA in SPM12 with age, sex, head motion, and adjusted HAMD score as covariates. For regions with significant differences, we further performed *post hoc* region of interest (ROI) analysis among the three groups to investigate whether these differences were specific to insomnia or MDD. In addition, the two-sample t test in SPM12 was used to investigate group comparisons in SCN FC maps between the whole MDD and HC groups with age, sex and head motion as covariates. The above significance threshold was set as *p* < 0.005 at the voxel level and false discovery rate (FDR)-corrected *p* < 0.05 at the cluster level.

#### 2.6.3. Correlation analysis

Furthermore, we performed partial correlation analyses to investigate the relationship between SCN FC changes and clinical symptom severity (total and factor scores of HAMD and HAMA) and subjective sleep quality (PSQI scores) in all MDD youths in SPSS19 (SPSS, Inc., Chicago, IL, United States) with age, sex, and head motion as covariates.

## 3. Results

### 3.1. Demographics and clinical characteristics

**Figure 1:**
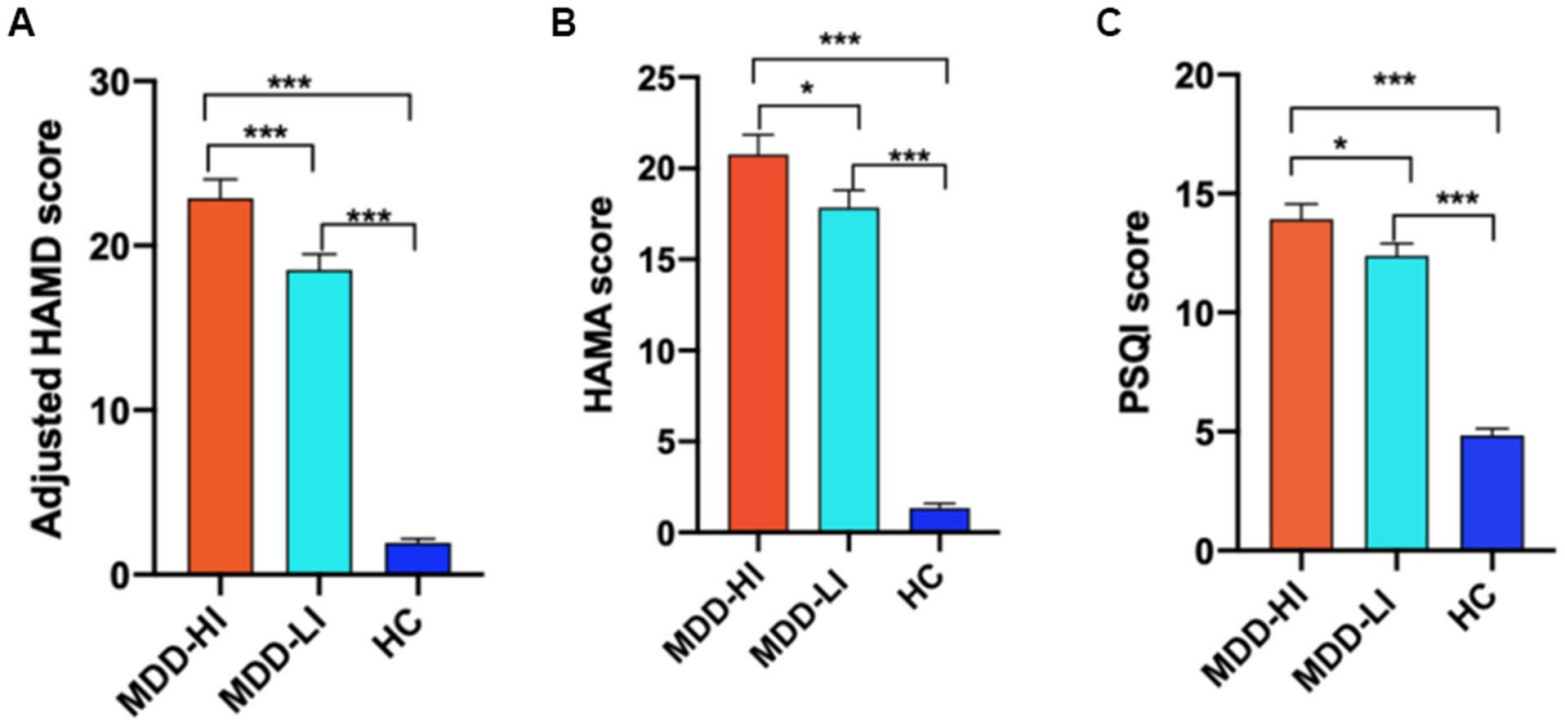
The adjusted HAMD, HAMA, and PSQI scores were compared among the three groups (MDD-HI, MDD-LI, and HC). The results showed that there were significant differences in the adjusted HAMD scores **(A)**, HAMA scores **(B)**, and PSQI scores **(C)** among the three groups. HAMD, Hamilton Rating Scale for Depression; Adjusted HAMD, HAMD scores after omission of the sleep subscale; HAMA, Hamilton Rating Scale for Anxiety; PSQI: Pittsburgh Sleep Quality Index; MDD-HI, major depressive disorder with high levels of insomnia; MDD-LI, major depressive disorder with low levels of insomnia; HC, healthy control. A single asterisk indicates *p* < 0.05, and double asterisks indicate *p* < 0.01. Three asterisks indicate *p* < 0.001.

The demographic details and clinical characteristics of MDD patients and HCs in the study are provided in Table 1. There were no significant differences in sex, years of education or head motion among the three groups (*p* > 0.05). Compared with the MDD-HI group, the MDD-LI group was older (*p* < 0.05). There were significant differences in HAMD, HAMA, and PSQI scores among the three groups (*p* < 0.0001). *Post hoc* analyses showed that compared with the MDD-HI group, the MDD-LI group showed lower scores on the HAMD, adjusted HAMD, HAMA, and PSQI (*p* < 0.05) (Figure 1). Both depressed patients exhibited significantly higher scores on the HAMD, HAMA, and PSQI (*p* < 0.05).

**Table 1:** Demographic and clinical characteristics of all participants.

Clinical data	MDD-HI (*n* = 29)	MDD-LI (*n* = 39)	HC (*n* = 43)	Statistics	*p* value	MDD-HI vs. MDD-LI	MDD-HI vs. HC	MDD-LI vs. HC
						*p* value	*p* value	*p* value
Age (years)	14.07 ± 1.44	15.18 ± 1.45	14.63 ± 1.90	3.854	0.024^b^	0.007	0.158	0.131
Sex (F/M)	27/6	38/12	25/33	3.618	0.164^a^			
Education (years)	8.10 ± 1.37	8.90 ± 1.52	8.63 ± 1.68	2.061	0.132^b^			
FD	0.07 ± 0.04	0.07 ± 0.03	0.07 ± 0.03	0.071	0.931^b^			
**HAMD-24**								
HAMD total score	27.21 ± 6.08	20.33 ± 6.10	2.16 ± 1.88	260.416	<0.001^b^	<0.001	<0.001	<0.001
Adjusted HAMD	22.90 ± 6.12	18.54 ± 5.91	1.93 ± 1.68	201.414	<0.001^b^	<0.001	<0.001	<0.001
Anxiety/somatization	5.45 ± 1.86	4.59 ± 2.11	1.14 ± 0.99	69.467	<0.001^b^	0.04	<0.001	<0.001
Weighting loss	0.90 ± 0.86	0.69 ± 0.80	0.07 ± 0.26	15.774	<0.001^b^	0.213	<0.001	<0.001
Cognitive disturbance	6.72 ± 2.62	5.21 ± 1.88	0.28 ± 0.50	137.369	<0.001^b^	0.001	<0.001	<0.001
Diurnal variation	0.52 ± 0.51	0.74 ± 0.88	0.02 ± 0.15	15.923	<0.001^b^	0.121	0.001	<0.001
Retardation	4.62 ± 1.80	4.02 ± 1.98	0.16 ± 0.43	98.619	<0.001^b^	0.112	<0.001	<0.001
HAMD-S	4.31 ± 0.71	1.79 ± 1.17	0.23 ± 0.53	198.88	<0.001^b^	<0.001	<0.001	<0.001
Hopelessness	3.39 ± 1.90	2.84 ± 1.72	0.16 ± 0.45	55.845	<0.001^b^	0.029	<0.001	<0.001
**HAMA-14**								
HAMA total score	20.76 ± 5.88	17.85 ± 6.00	1.35 ± 1.59	186.415	<0.001^b^	0.014	<0.001	<0.001
Somatic anxiety	9.28 ± 3.34	7.36 ± 3.36	0.12 ± 0.39	128.1	<0.001^b^	0.004	<0.001	<0.001
Psychic anxiety	11.48 ± 4.0	10.49 ± 358	1.23 ± 1.43	131.186	<0.001^b^	0.19	<0.001	<0.001
**PSQI**								
PSQI total score	13.93 ± 3.37	12.38 ± 3.23	4.84 ± 1.93	112.471	<0.001^b^	0.028	<0.001	<0.001
sleep quality	2.31 ± 0.85	2.26 ± 0.79	0.95 ± 0.72	38.192	<0.001^b^	0.778	<0.001	<0.001
sleep latency	2.24 ± 0.79	2.31 ± 0.66	1.0 ± 0.95	32.567	<0.001^b^	0.74	<0.001	<0.001
sleep duration	2.31 ± 0.66	1.87 ± 1.03	0.21 ± 0.47	81.688	<0.001^b^	0.02	<0.001	<0.001
Sleep efficiency	2.0 ± 1.25	1.28 ± 1.19	0.60 ± 0.79	14.811	<0.001^b^	0.007	0.009	<0.001
sleep disturbance	1.90 ± 0.82	1.77 ± 0.78	0.91 ± 0.57	21.896	<0.001^b^	0.469	<0.001	<0.001
use of sleeping medication	0.79 ± 1.05	0.67 ± 0.96	0 ± 0	11.509	<0.001^b^	0.509	<0.001	<0.001
daytime dysfunctions	2.31 ± 1.0	2.18 ± 0.82	1.16 ± 0.81	20.188	<0.001^b^	0.541	<0.001	<0.001

a *p* values were obtained using the chi-square test.

b *p* values were obtained via one-way analysis of variance (ANOVA).

HC, healthy control; MDD-HI, major depressive disorder with high levels of insomnia; MDD-LI, major depressive disorder with low levels of insomnia; HAMD, Hamilton Rating Scale for Depression; Adjusted HAMD, HAMD scores after omission of the sleep subscale; HAMD-S, HAMD sleep subscale; HAMA, Hamilton Rating Scale for Anxiety; FD, framewise displacement, PSQI, Pittsburgh Sleep Quality Index.

In the whole MDD group, the PSQI total score was significantly positively correlated with the HAMD-S score (*r* = 0.292, *p* = 0.016), HAMD total score (*r* = 0.256, *p* = 0.035) and HAMA total score (*r* = 0.270, *p* = 0.026). The PSQI-sleep duration factor score was positively correlated with the HAMA total score (*r* = 0.252, *p* = 0.038) and psychic anxiety (*r* = 0.252, *p* = 0.038).

### 3.2. Comparison of SCN FC maps

**Figure 2:**
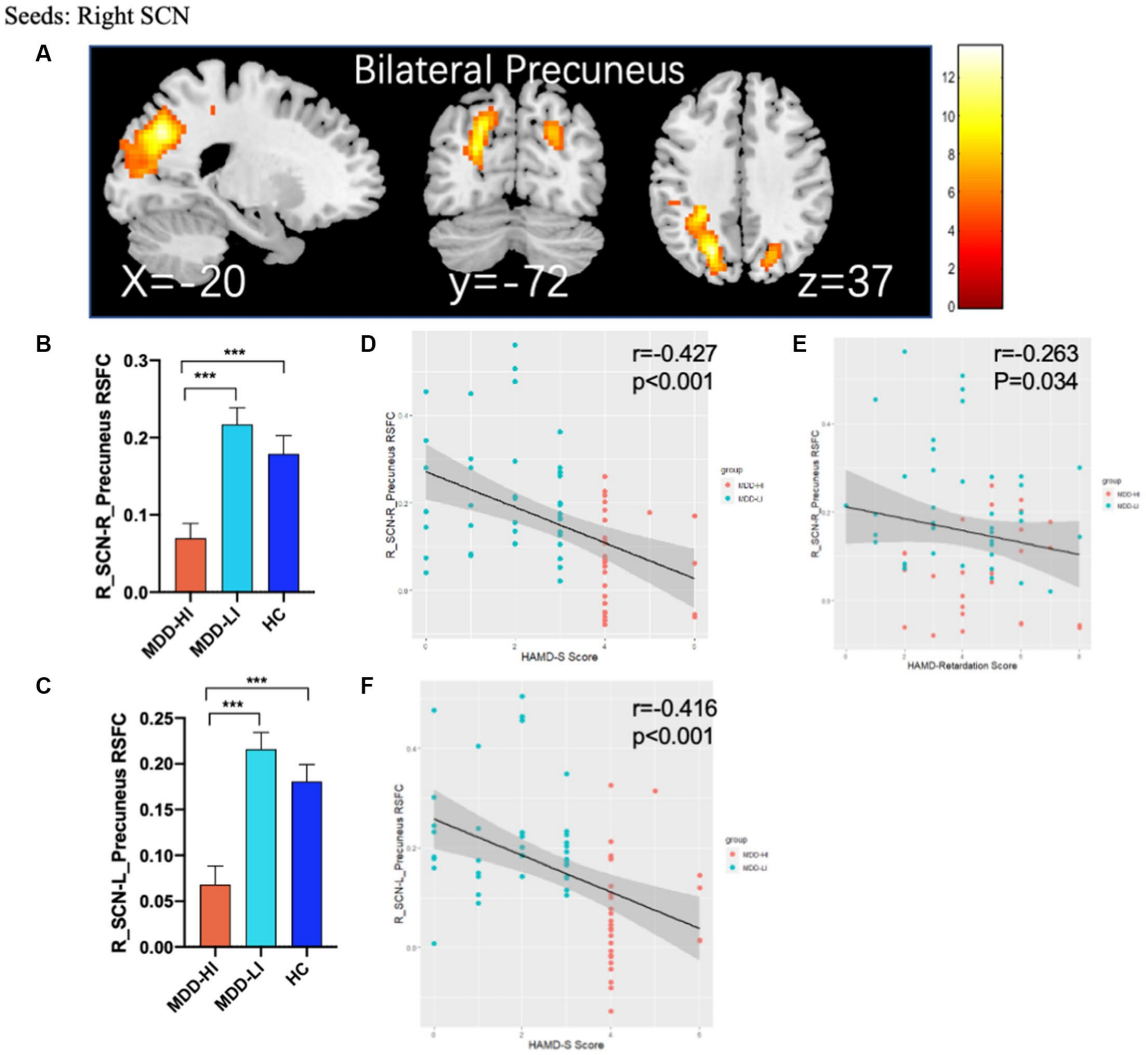
Abnormal rs-FC between the SCN and other regions and partial correlation between altered brain function and clinical scores. **(A)** ANOVA with age, sex, head motion, and adjusted HAMD score as covariates showed that there were significant differences in FC between the right SCN and bilateral precuneus in the adolescent MDD-HI, MDD-LI, and HC groups. The results of *post hoc* analyses on these areas are shown in **(B,C)** and showed decreased FC in the adolescent depression with high insomnia group. **(D–F)** Partial correlation between altered brain function and clinical scores. The horizontal axis represents the clinical scores of patients with MDD, while the vertical axis represents the FC values between the right SCN and bilateral precuneus. The orange triangles represent patients with MDD-HI, and blue triangles represent patients with MDD-LI. HAMD-S, HAMD sleep subscale; MDD-HI, major depressive disorder with high levels of insomnia; MDD-LI, major depressive disorder with low levels of insomnia; HC, healthy control; SCN, suprachiasmatic nucleus. Three asterisks indicate *p* < 0.001.

Compared to HCs and MDD-LI youths, MDD-HI youths showed decreased FC between the right SCN and the bilateral precuneus (Figure 2; Table 2). However, there was no significant difference between the MDD-LI and HC groups. In addition, there was no significant group difference in left SCN FC among the three groups.

**Table 2:** Brain regions with ROI-based functional connectivity differences among the three groups.

Seed	Group	Regions	L/R	Cluster size	Peak (MNI) X Y Z	Statistics	P (FDR-corr)
R_SCN	ANOVA	PCUN	L	977	−27, −39, 45	16.11	<0.001
		PCUN	R	274	21, −72, 33	9.34	0.01
	MDD-HI < MDD-LI	PCUN	L	977	−27, −39, 45	27.13	<0.001
	MDD-HI < HC	PCUN	L	977	−27, −39, 45	24.25	<0.001
	MDD-HI < MDD-LI	PCUN	R	274	21, −72, 33	20.19	<0.001
	MDD-HI < HC	PCUN	R	274	21, −72, 33	17.405	<0.001

SCN, suprachiasmatic nucleus; MDD-HI, major depressive disorder with high levels of insomnia; MDD-LI, major depressive disorder with low levels of insomnia; HC, healthy control; MNI, Montreal Neurological Institute; PCUN, precuneus; L, left; R, right; FDR, false discovery rate.

There were no significant group differences in SCN FC maps between the whole MDD and HC groups.

### 3.3. Correlation analysis

The right SCN-bilateral precuneus connectivity was negatively correlated with HAMD-S scores in the whole MDD group (*p* < 0.001) after controlling for the effects of age, sex and head motion. Moreover, there was also a negative correlation between right SCN-right precuneus connectivity and retardation scores in the whole MDD group (*p* = 0.034, *r* = −0.263) after controlling for the effects of age and sex (Figure 2). No significant correlation between SCN-precuneus connectivity and other factors of HAMD, HAMA and PSQI scores and clinical severity were detected.

## 4. Discussion

To the best of our knowledge, this is the first study to investigate the role of the SCN in sleep symptoms in adolescents with MDD. Compared to MDD-LI patients and HCs, adolescent MDD-HI patients demonstrated significantly decreased FC between the right SCN and bilateral precuneus. More importantly, the symptoms of insomnia were negatively correlated with FC of the right SCN and bilateral precuneus. Our results suggest that SCN-precuneus connectivity may contribute to sleep quality in adolescents with MDD. Our findings offer a neural mechanism related to the association between sleep symptoms and MDD.

The clinical features of the three subgroups confirmed the close association and comorbidities between MDD and sleep symptoms. In the present study, all adolescents with MDD had a PSQI score greater than 5, which indicates that a subject has severe difficulties in at least 2 domains or moderate difficulties in more than 3 domains (32). The HAMD, HAMD-S and HAMA scores were positively correlated with the PSQI scores in depressed adolescents. This implies that the worse the sleep quality, the more serious the depressive and anxiety symptoms. Sleep is thought to play a critical role in brain development and function (34–36), and insomnia can lead to cognitive decline and a range of physical symptoms (37, 38), and cognitive control is present throughout the emotional regulation process (39). In the current research, adolescents with MDD with high insomnia showed more severe depression, more physical symptoms, reduced cognitive function, and feelings of hopelessness. The onset of puberty is correlated with a time delay in the circadian system, such as delayed sleep (40, 41). A recent meta-analysis showed that depressed adolescents have more awakenings in bed, and longitudinal analyses suggest that sleep disturbances may predict depression (9). Therefore, we speculate that insomnia in teenagers may be the cause or underlying factor of some physical and psychiatric symptoms, eventually leading to depression.

The SCN coordinates the circadian rhythm through neural and endocrine regulation (15). The difficulty of falling asleep, the duration and structure of sleep are all regulated by the circadian rhythm phase of the central pacemaker located in the SCN (42). Abnormalities in SCN function are often observed in patients with sleep disorders. Patients with chronic insomnia have abnormal FC between the SCN and the frontal cortex, locus coeruleus and raphe nuclei (43). The SCN and default mode network (DMN) show synchrony in regulating the arousal state of the system (44). After sleep deprivation, the FC of DMN weakened (45). The abnormal FC between SCN and DMN may be the underlying pathophysiological mechanism of sleep-wake disorders in delirium patients (46).

As a key node in the posteromedial cortex, the precuneus is involved in a variety of complex functions, such as visuospatial imagery, self-processing, episodic memory retrieval and consciousness (47). Activation of the precuneus correlates with rumination (48). Furthermore, the precuneus is the structural core of the DMN, whose dysfunction has been widely reported in depression. The intrinsic FC between striatum and DMN is associated with the severity of depression in adolescent MDD patients (49). Increased DMN FC was associated with decreased HAMD scores in MDD (50). Recent studies have suggested that the precuneus plays a pivotal role between depression and sleep quality. For example, in individuals with MDD, sleep quality was negatively correlated with precuneus fractional amplitude of low-frequency fluctuation (fALFF) and gray matter volume (GMV) (51). Increased FC in the precuneus was associated with sleep quality and depression scores in healthy adults (52). MDD patients with more severe insomnia showed decreased surfaces in multiple frontoparietal cortical areas (53). Higher volume brain regions, including the precuneus, were correlated with longer sleep duration (54). In addition, FC between the right precuneus and the dorsal lateral prefrontal cortex or posterior cingulate cortex increased after antidepressant treatment and was positively correlated with N3 sleep (slow wave sleep) (55). In conclusion, the precuneus may play a role in the relationship between depression and sleep as an important neuroanatomy.

The light–dark (LD) cycle, as a zeitgeber (from the German for time giver), is involved in visual processing and plays an important role in SCN circadian synchrony (56). One possible explanation is that abnormal FC between the SCN and vision-related areas was a characteristic of sleep-related circadian disruption in depression. In the current study, we observed a significant reduction in FC between the right SCN and the bilateral precuneus (primary visual cortex center) (57). Previous studies have suggested that MDD patients have abnormal FC in brain regions closely related to visual processing, including the calcarine sulcus, lingual gyrus, and fusiform gyrus, which is related to sleep quality (23, 26). Current evidence suggests that FC between the SCN and visual cortex is involved in the maintenance of circadian rhythms in depressed patients.

Furthermore, the decreased FC between the SCN and the precuneus in MDD-HI might be explained by GABA (γ-amino butyric acid), which is the most abundant inhibitory neurotransmitter in the SCN (19). A recent review suggests that GABA may affect circadian rhythms by mediating oscillatory coupling of SCN circadian rhythms as well as improving circadian output rhythms. Previous studies have shown that GABA may mediate task-induced activity along the midline structure of the cerebral cortex (58) and is significantly associated with DMN deactivation where the precuneus is the main DMN hub (59). However, as we did not measure GABA levels in the current study, it is difficult to reach a definitive conclusion. However, we believe it is a field worth exploring.

The present study has several limitations. First, self-report questionnaires were used to measure sleep quality. These scales only generally reflect the quality of sleep or the symptoms of insomnia but cannot monitor changes in the sleep cycle or rhythm. Second, the cross-sectional design makes it difficult to draw firm conclusions about the underlying mechanism of the significant association between sleep quality and depressive symptoms in adolescents. Future studies need to use more objective tools, such as polysomnography, to measure sleep changes and incorporate network analysis in combination with neurotransmitter or hormone level tests to clarify the exact mechanism of the current findings. Moreover, we emphasized that the causal relationship between functional connectivity and sleep is an important topic for further exploration. Thus, longitudinal studies are needed to elucidate the causal relationship between depressive symptoms and sleep quality.

In summary, we are the first to report that the connectivity between the right SCN and bilateral precuneus was more impaired in adolescents MDD-HI than in adolescents MDD-LI and HCs. The results of this study provide a preliminary exploration for interventions based on imaging changes related to insomnia symptoms in adolescents with MDD. The altered FC between the SCN and precuneus underlines the neural mechanism of sleep-related symptoms in depressive adolescent MDD patients and demonstrates potential targets for personalized treatment strategies.

## Data availability statement

The original contributions presented in the study are included in the article/supplementary material, further inquiries can be directed to the corresponding authors.

## Ethics statement

The studies involving human participants were reviewed and approved by The Ethics Committee of the Third People’s Hospital of Mianyang. Written informed consent to participate in this study was provided by the participants’ legal guardian/next of kin.

## Author contributions

GH and XQH designed the study. RF, YL, LHZ, and GH participated in the patient recruitment. WB and ZZ performed the MRI preprocessing and quality assessment. LC, RF, YG, and KL performed the data analyses and statistics. LC and RF wrote the frist manuscript. XYH, HL, LQZ, and XQH critically reviewed and revised it for important intellectual content. All authors contributed to the article and approved the submitted version.

## Funding

This study was supported by grants from the Natural Science Foundation of Sichuan Province (2022NSFSC0052) and the Clinical and Translational Research Fund of the Chinese Academy of Medical Sciences (2021-I2M-C&T-B-097).

## Conflict of interest

The authors declare that the research was conducted in the absence of any commercial or financial relationships that could be construed as a potential conflict of interest.

## Publisher’s note

All claims expressed in this article are solely those of the authors and do not necessarily represent those of their affiliated organizations, or those of the publisher, the editors and the reviewers. Any product that may be evaluated in this article, or claim that may be made by its manufacturer, is not guaranteed or endorsed by the publisher.

## Footnotes

^1^ http://www.restfmri.net

^2^ http://restfmri.net/forum/index.php?q=rest
